# Competition's Role in Shaping Cryptic Genetic Variation

**DOI:** 10.1111/ede.70033

**Published:** 2026-03-01

**Authors:** C. Hal Terry, Dante J. Nesta, Lucia Caluseriu, Meredith Pearson, Cristina C. Ledón‐Rettig

**Affiliations:** ^1^ Department of Biology Indiana University Bloomington Bloomington Indiana USA

**Keywords:** amphibian, developmental plasticity, genetic accommodation, novelty, plasticity‐first evolution, polyphenism, tadpole

## Abstract

Cryptic genetic variation—heritable genetic variation that is only expressed under stressful or novel environments—can potentially fuel the evolution of novel traits. While previous work has demonstrated that novel environments can expose cryptic genetic variation, whether and how multiple environments interact to shape such variation in natural populations is poorly understood. To determine how multiple environments may modulate cryptic genetic variation, we used tadpoles of the Eastern spadefoot, *Scaphiopus holbrookii* (*Sc. holbrookii*). Species of *Scaphiopus* have previously been used as outgroups to the genus *Spea*, which has evolved a novel carnivorous tadpole morph specialized for a shrimp diet. Here, we assess whether shrimp‐induced cryptic genetic variation in *Sc. holbrookii* tadpole traits varies as a function of conspecific competition. Across all traits measured, we found that shrimp‐induced cryptic genetic variation only occurred under specific competitive conditions. Specifically, the shrimp diet revealed cryptic genetic variation in body size, gut length, and jaw area when tadpoles experienced high intraspecific competition. Surprisingly, across these same traits, the shrimp diet suppressed the expression of heritable variation under low competition, suggesting that moderately stressful conditions can limit the expression of heritable variation. In contrast to the other traits, the expression of heritable variation in tadpole tail depth was largely unaffected by diet or competition. Together, our results indicate that interacting environmental factors jointly modulate how and in what traits cryptic genetic variation may be expressed, thereby affecting its potential to drive novel trait evolution.

## Graphical abstract

Novel and stressful environments can lead to the expression of previously cryptic genetic variation. Using spadefoot tadpoles, we show that the expression of cryptic genetic variation depends on interacting environmental factors, such as diet and competition, suggesting that it contributes to evolutionary change only under specific environmental conditions.

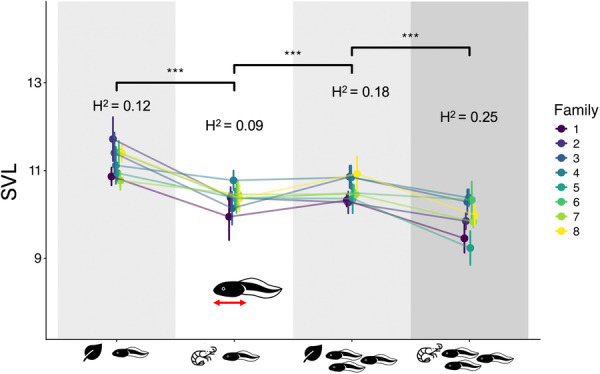

## 1 Introduction

Novel traits often confer advantageous functions to their bearers but lack obvious counterparts in closely related species (Muller and Wagner 1991). One central question in evolutionary biology is how such traits arise. For novel trait evolution to proceed, there must be standing heritable variation upon which selection can act. Yet the mechanisms by which such variation is expressed across variable environments are not fully understood.

The expression of heritable variation can change across environments due to phenotypic plasticity, the ability of organisms to alter their phenotypes in response to environmental change (West‐Eberhard 2003). Importantly, genotypes differ in the extent to which they enable trait plasticity, leading to differing amounts of heritable trait variation being expressed across environments (Gibson and Dworkin 2004). Particularly novel or stressful environments can reveal previously unexpressed, or “cryptic,” genetic variation, increasing the heritable variation available for selection (Paaby and Rockman 2014). If this phenotypically expressed variation is favored by natural selection, it can facilitate adaptation and even fuel the evolution of novel traits (West‐Eberhard 2003; Paaby and Rockman 2014). Indeed, a growing number of studies support the role of cryptic genetic variation in fueling the evolution of novel traits in both laboratory (Suzuki and Nijhout 2006) and natural populations (Ledón‐Rettig et al. 2010a; Berger et al. 2011).

While these studies demonstrate that cryptic genetic variation can be phenotypically expressed in novel or stressful environments, it remains unclear how it manifests under more complex genetic and environmental interactions. In particular, higher‐order interactions between genes and environments may result in phenotypic variation otherwise unseen when considering only pairwise (G × E) interactions (Weinreich et al. 2013; Schmidlin et al. 2025). For example, epistatic (G × G) fitness outcomes in *Arabidopsis thaliana* plants vary across different environments (i.e., G × G × E) (Kerwin et al. 2017). Similarly, growth in genetically divergent populations of *Manduca sexta* hornworms depends on the interacting effects of temperature and a novel diet (i.e., G × E × E) (Diamond and Kingsolver 2012; Westneat et al. 2019). As natural populations can simultaneously experience novel and stressful environments, understanding how these combinations can encourage or hinder the expression of cryptic genetic variation in natural populations may provide insight into the evolution of novel traits.

**Figure 1:**
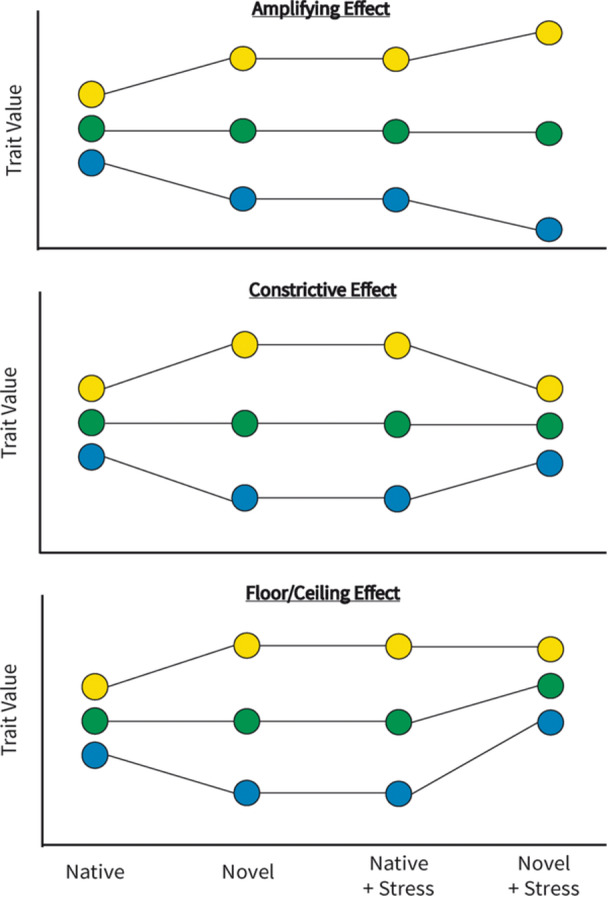
Predicted patterns of expressed genetic variation under interacting novel and stressful environments. Different colors represent the mean trait values of different hypothetical genotypes. Labels across the *x*‐axis represent different hypothetical environments in which those traits are expressed. [Color figure can be viewed at wileyonlinelibrary.com]

We can make several predictions about how stressful environments may shape the expression of cryptic genetic variation revealed under novel conditions (Rowiński and Rogell 2017). If additional stress amplifies the stress introduced by the novel environment, it could further disrupt canalization mechanisms, thereby exposing more cryptic genetic variation (Rutherford 2000) (Figure 1). Alternatively, increased environmental stress, such as nutritional deficiency, may hinder growth and trait expression across genotypes, thereby constricting the expression of cryptic genetic variation (Hoffmann and Merilä 1999). In this scenario, we might expect lower genetic variance in environments that are both novel and stressful (Figure 1). Further, under heightened stress, trait expression may approach developmental or physiological limits—phenotypic “floors” or “ceilings”—that constrain phenotypes regardless of genetic background (Gomez‐Mestre et al. 2008) (Figure 1). Under such conditions, genetic variance could be diminished as traits converge on these boundaries.

Here we address how interacting environments reveal cryptic genetic variation across traits in North American spadefoot toads (Ledón‐Rettig et al. 2010a; Levis et al. 2020). In the genus *Spea*, tadpoles exhibit a developmental polyphenism, producing either a typical omnivore morph that consumes detritus or a derived carnivore morph that consumes and is induced by shrimp diet—a diet that is novel when considering *Spea's* ancestors (Pomeroy 1981). The novel *Spea* carnivore morph exhibits a suite of integrated, specialized traits, including pronounced jaw muscles and a shortened gut, optimized for consuming shrimp (Pomeroy 1981). Like all other anuran (frog) taxa, *Spea's* sister genus *Scaphiopus* lacks this polyphenism. *Scaphiopus* develops only as the ancestral omnivore morph, making it an appropriate outgroup for understanding ancestral plasticity and the expression of cryptic genetic variation (Ledón‐Rettig et al. 2010a). Although *Scaphiopus* does not induce the complex and integrated carnivore morph, a shrimp diet induces variation in traits that are highly modified in the *Spea* carnivore morph, both developmentally and evolutionarily. Specifically, *Scaphiopus* tadpoles show increased heritable variation in these traits following exposure to a novel shrimp diet, that is, revealing cryptic genetic variation (Ledón‐Rettig et al. 2010a; Levis et al. 2020; Pomeroy 1981; Ledón‐Rettig et al. 2010a). If such continuous, diet‐induced variation across traits was co‐selected in ancestral *Spea*, it could have been refined and brought under shared genetic control, ultimately giving rise to a functionally integrated ecomorph (Cheverud 1996; Wagner 1996; Murren 2012).

We hypothesize that the ecological stressor of competition interacts with the novel shrimp diet to shape expressed genetic variation in *Scaphiopus* tadpoles. We focused on competition as an ecological stressor for two reasons: (i) competition increases heritable variation in another *Scaphiopus* tadpole trait, brain gene expression (Nesta and Ledón‐Rettig 2025), and (ii) in nature, *Spea* carnivore morphs are most often found in ponds that are both shrimp‐rich and crowded with conspecifics (Martin and Pfennig 2010). If similar environmental interactions shaped expressed genetic variation in the ancestors of *Spea*, they might have facilitated the evolution of *Spea'*s novel polyphenism. Here, we tested whether conspecific competition interacts with the novel diet type to influence the expression of cryptic genetic variation in nonpolyphenic *Scaphiopus holbrookii* (*Sc. holbrookii*) tadpoles.

## 2 Methods

### 2.1 Experimental Design

To determine the effects of competition on shrimp‐induced cryptic genetic variation, we reared families of *Sc. holbrookii* tadpoles under different dietary and competitive environments. In Greene County, Indiana, we collected eggs from eight clutches that were at least 1 m apart, such that each clutch likely represented a different family. We transported these eggs to Indiana University and reared them in Novaqua‐treated (Kordon) and aged (allowed to sit overnight) water. Prior to hatching, all embryos experienced common garden conditions; that is, each family was raised separately in the same‐sized containers, in the same temperature‐controlled (~25°C) room. We placed randomly selected recently hatched tadpoles from each family into clear plastic microcosms (15 × 9 × 10 cm^3^) with controlled diet and competition levels; that is, each microcosm contained only siblings. We fed tadpoles either Hikari fish pellets as the ancestral detritus diet or brine shrimp (*Artemia*) as the novel diet, ad libitum. Because the Hikari fish food contains animal, yeast, and algal material, it captures the broad range of food items that detritus‐consuming omnivores eat in nature. We modulated competition by distributing either 3 or 8 tadpoles per microcosm for low and high competition treatments, respectively. These densities are at the low and high ends of densities of spadefoot tadpoles observed in natural ponds (4–11 per liter) (Pfennig et al. 1991; Bazazi et al. 2012). Although tadpoles in the high‐competition treatment did not experience lower resource levels (because they were fed ad libitum), they experienced higher perceived competition through one or a combination of olfactory, visual, or tactile cues (Rot‐Nikcevic et al. 2005; Fraker et al. 2009). Indeed, previous work has shown that increased conspecific density can elevate corticosterone levels, a stressor‐responsive hormone, even if food levels per tadpole are the same (Hayes 1997; Glennemeier and Denver 2002).

In total, we used 256 microcosms representing a sample size of *n* = 64 per diet‐competition treatment and sampled the second‐largest tadpole from each microcosm. To ensure consistent diet conditions, we did not include tadpoles from microcosms where cannibalism occurred. We reared tadpoles until Gosner Stage 33, after which we euthanized and preserved them in buffered formalin.

### 2.2 Trait Measurements

We weighed each tadpole and measured its body size as snout–vent length (SVL), along with jaw muscle area, tail depth, and gut length. While we measured tadpole tail depth with digital calipers, we photographed and digitally measured all other traits with Fiji software (Schindelin et al. 2012). To prepare the gut and jaw muscles for measurements, we first dissected each tissue. We measured each trait with a 10 mm scale bar as a reference. We size‐corrected gut length, jaw area, and tail depth by regressing these trait measurements against a tadpole's respective body size—within diet and density treatments—and then calculated common within‐group residuals.

### 2.3 Statistical Analyses

We estimated levels of genetic variance in *Sc. holbrookii* tadpole traits across different diet and density levels with linear mixed modeling. To do this, we built a linear mixed model for each morphological trait via the glmmTMB package (version 1.1.13) in R (version 4.5.0), including microcosm as a random intercept, as well as different density and diet treatments as a random slope among families (Brooks et al. 2017; R Core Team 2025). We also adjusted the dispersion formula to allow different variances for each treatment, rather than assuming a common environmental variance (dispformula = ~0 + diet:density). Our resulting model was:

Responsetrait=1+diet∗density(0+diet:density|family)+(1|Microcosm).

We specifically examined body size, residual jaw area, residual tail depth, and residual gut length as response variables, all of which have previously been reported as responsive to novel or stressful environments in anuran tadpoles (Relyea 2004; Ledón‐Rettig et al. 2010a).

We used the variance components from each trait's linear mixed model to estimate broad‐sense heritability, which predicts each trait's potential response to selection (Roff 2012). First, we extracted family variance under each diet‐density treatment and residual variance from each model. Using these variance estimates, we used the following equation to estimate the heritability for each trait,

$$H^{2}=\frac{2\;*\;V_{\mathrm{AF}}}{V_{\mathrm{AF}}+V_{M}+V_{\mathrm{AP}}}$$

where $V_{\mathrm{AF}}$ represents among‐family variance, $V_{M}$ as variation attributed to each microcosm, $V_{\mathrm{AP}}$ represents within‐family variance, and $H^{2}$ is broad‐sense heritability (Roff 2012). To compare heritabilities across treatments, we bootstrapped our linear mixed models using the R function *bootmer* via the lme4 R package (version 1.1‐37) with 10000 permutations (Bates et al. 2015). From this, we generated confidence intervals for heritability estimates across diet and density treatments for each trait. We used nonparametric Wilcoxon tests to compare distributions of trait heritabilities across treatments for each trait; significantly higher heritability under the shrimp (novel) diet was interpreted as the expression of cryptic genetic variation (Levis et al. 2020). We applied a Bonferroni correction to account for multiple tests (*n* = 8), adjusting the significance threshold of our Wilcoxon tests to *p* < 0.00625. Point estimates and 95% confidence intervals for heritability were also generated (Table S1). We also calculated the coefficient of genetic variation for each trait, though we used the original (uncorrected) morphological measures (Houle 1992) (Tables S2 and S3). Lastly, we generated models for the heritability of body size and residual tail depth with all individuals per microcosm (Figure S1).

## 3 Results

**Figure 2:**
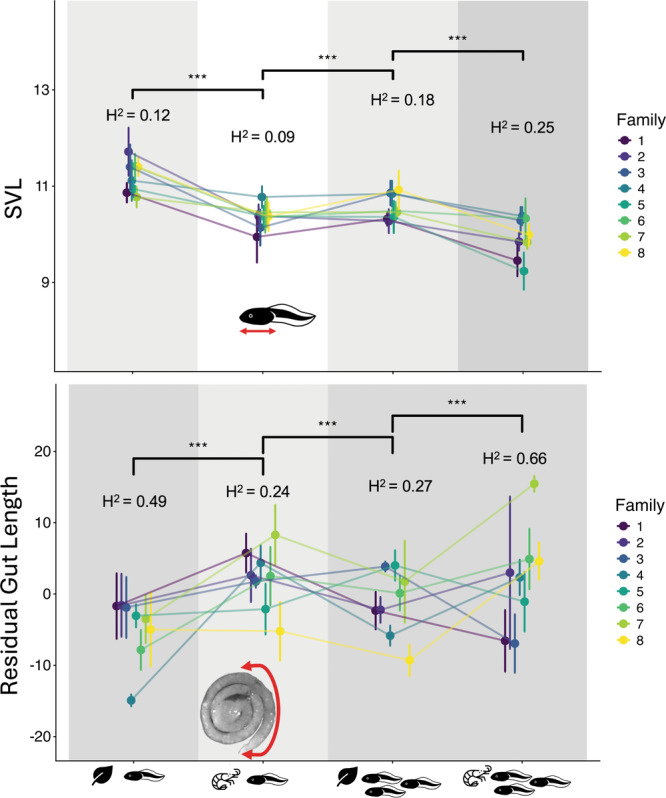
Shrimp‐induced cryptic genetic variation is dependent on trait and competitive environment. Reaction norms of family mean trait values and associated standard errors are plotted for snout–vent length (top) and residual gut length (bottom). The estimated heritability for each family/group was calculated from genetic and environmental variance, indicated by the background shading (white: 0–0.11, light gray: 0.12–0.24, and darker gray: 0.25 and above). Significant (*p* < 0.00625) differences of heritability between treatments are represented by the brackets and asterisks (***) above the compared groups. [Color figure can be viewed at wileyonlinelibrary.com]

**Figure 3:**
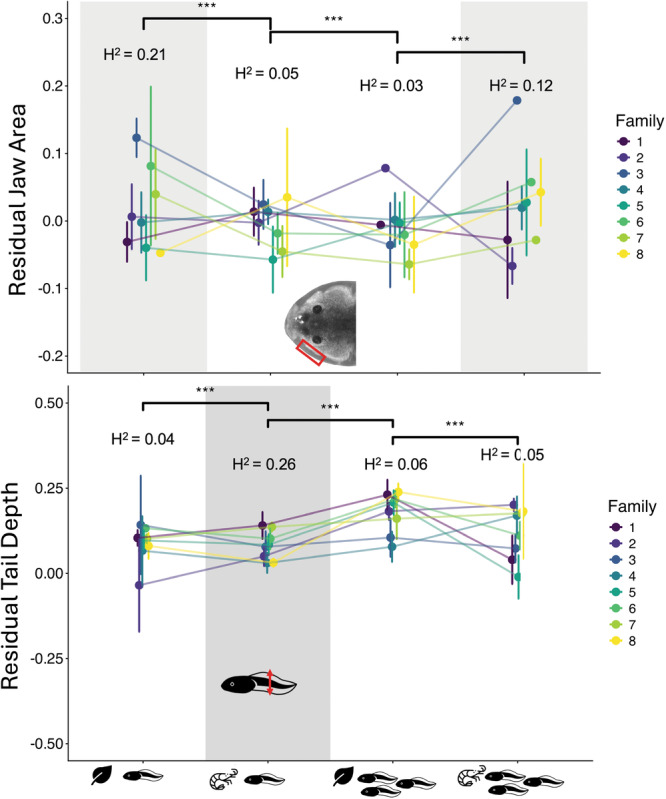
Shrimp‐induced cryptic genetic variation is dependent on trait and competitive environment. Reaction norms of family mean trait values and associated standard errors are plotted for residual jaw area (top) and residual tail depth (bottom). The estimated heritability for each family/group was calculated from genetic and environmental variance, indicated by the background shading (white: 0–0.11, light gray: 0.12–0.24, and darker gray: 0.25 and above). Significant (*p* < 0.00625) differences of heritability between treatments are represented by the brackets and asterisks (***) above the compared groups. [Color figure can be viewed at wileyonlinelibrary.com]

Shrimp‐revealed cryptic genetic variation in *Sc. holbrookii* tadpole traits varied across density levels (Figures 2 and 3). For body size, residual gut length, and residual jaw area, the shrimp diet decreased heritability under low competition but increased it under high competition (Table 1 and Figure 2). In contrast, for residual tail depth, the shrimp diet increased heritability under low competition, but decreased heritability under high competition (Table 1 and Figure 3), although heritability of this trait was generally low across treatments.

**Table 1:** Shrimp‐induced cryptic genetic variation is dependent on trait and competitive environment.

	Low			High		
Trait	Detritus	Shrimp	*p*	Detritus	Shrimp	*p*
SVL	0.12	0.09	< 2.2e − 16	0.18	0.25	< 2.2e − 16
GL	0.49	0.24	< 2.2e − 16	0.27	0.66	< 2.2e − 16
JA	0.21	0.05	< 2.2e − 16	0.03	0.12	< 2.2e − 16
TD	0.04	0.26	< 2.2e − 16	0.06	0.05	< 2.2e − 16

*Note:* Heritability estimates derived from bootstrapped variances were extracted from a linear mixed model (10,000 permutations) and Wilcoxon test comparisons between diets across density levels. Yellow *p* values indicate higher heritability under shrimp diets (revealing CGV), while blue values represent higher heritability under detritus diets. Bonferroni corrections were used to account for multiple tests (*n* = 8), resulting in a new alpha value of *p* < 0.00625.

## 4 Discussion

Cryptic genetic variation is thought to fuel novel trait evolution in the face of environmental change (Paaby and Rockman 2014), yet how its expression is shaped by higher‐order interactions among genes and the environment remains poorly understood. Here, we used *Sc. holbrookii* tadpoles to test whether the expression of cryptic variation in response to a novel diet is influenced by conspecific competition. Our results align with prior studies describing diet‐induced cryptic genetic variation in traits associated with the novel *Spea* polyphenism (gut length, jaw area, and body size) (Ledón‐Rettig et al. 2010a; Levis et al. 2020). However, we further demonstrated that the expression of this cryptic genetic variation only occurs under a competitive environment. In fact, consuming shrimp reduced heritability in these traits under low competition. One trait, tail depth, was largely unaffected by either diet or competition. Together, our results suggest that higher‐order interactions (i.e., G × E × E) modulate the expression of cryptic genetic variation in a trait‐specific manner.

Most traits we measured responded similarly to diet and intraspecific competition: the highest levels of expressed heritable variation occurred when tadpoles were fed shrimp under high competition or detritus under low competition. On the one hand, the high levels of expressed heritable variation when tadpoles are fed shrimp under high competition suggest that the expression of cryptic genetic variation depends on experiencing both ecological factors simultaneously, which together generate a stress level not achieved by each factor alone (“Amplifying Effects”). This is supported by the observation that the mean body size of *Sc. holbrookii* tadpoles was lowest under these combined conditions. Further, previous studies have shown that both novel diets (Ledón‐Rettig et al. 2010b) and competition (Glennemeier and Denver 2002) independently reduce body size and elevate corticosterone—a vertebrate hormone associated with stress—in tadpoles, consistent with a model in which combined ecological stressors trigger the expression of cryptic genetic variation. Moreover, cryptic genetic variation has been revealed in *Sc*. *couchii*, a sister species to *Sc. holbrookii*, by exposure to exogenous corticosterone (Ledón‐Rettig et al. 2010a). Together with previous studies, our results suggest that, for some traits, the expression of cryptic genetic variation depends on multiple environmental stressors acting in concert.

On the other hand, the high heritable variation observed across body size, gut length, and jaw area when tadpoles were fed detritus under low competition suggests that the expression of heritable variation also occurs readily under benign conditions. This pattern is consistent with the Constrictive Effects model, wherein the stress of a novel diet or competition limits growth and constrains trait variation (Newman 1988; Hoffmann and Merilä 1999). For example, in a previous study, *Sc. couchii* tadpoles exhibited reduced heritability in growth and developmental speed under pond‐drying conditions (Newman 1988). Unlike that study—where tadpole size and developmental period converged on minimum size values due to “Floor Effects”—our results show no evidence of such boundaries. Mean values of *Sc. holbrookii* body size, gut length, and jaw area when fed the novel shrimp diet (under low competition) were intermediate relative to their values when fed the detritus diet, even though the expressed heritable variation in these traits decreased. That the expression of cryptic genetic variation depended entirely on the competitive environment underscores the importance of considering multiple environmental factors and levels of environmental stress when evaluating the role of cryptic genetic variation in adaptation.

In addition to analyses of traits associated with the *Spea* polyphenism (body size, jaw area, and gut length), we also examined the heritability of tail depth across diet and density treatments. While heritability in tail depth increased with a novel shrimp diet under low competition (indicating some sensitivity to novel conditions), heritability estimates for this trait were consistently lower compared to those of other traits, across all environments. This limited response—in both heritable variation and mean trait value—to competition and diet is unlikely to reflect trait canalization, as plasticity in tadpole tail depth is common in many anuran species, including other spadefoots (Relyea 2004; Kelly et al. 2021). However, such plasticity is typically triggered by predator exposure or cues from injured conspecific. Taken together with the absence of known tail‐depth differences between the two *Spea* morphs, our findings suggest that diet and competition are not meaningful cues with respect to tail depth variation.

Our findings of differing heritability across diet and densities for *Sc. holbrookii* provide insight into the conditions that may have driven the evolution of the *Spea* tadpoles polyphenism. If the responses of *Sc. holbrookii* tadpole traits mirror the ancestral heritable variation of *Spea* tadpoles, then the evolution of the predatory morph likely occurred most rapidly when populations experienced environments rich in shrimp and intraspecific competitors. Notably, these are the very conditions that promote the induction of the novel carnivore morph in *Spea* (Pfennig and Murphy 2002). Additionally, other morphological, behavioral, or physiological traits may show differences in variation across diet and density treatments. For instance, behavioral variation, which can be heightened under environmental stress, may drive variation in competitive ability. This pattern has been observed in Trinidadian guppies (*Poecilia reticulata*) where aggression increases in a non‐native predator environment (Fischer et al. 2016). Likewise, physiological traits such as digestion efficiency, which is plastic in other anurans (*e.g*., *Rana temporaria*) (Liess et al. 2015), may also differ across these environments. If these aspects of *Sc. holbrookii* also mirror that of ancestral *Spea*, it may serve as a window into the evolution of the carnivorous behavioral polyphenism or ability to easily digest the novel shrimp. Future studies assessing cryptic genetic variation in *Scaphiopus* should integrate behavioral and physiological traits, given their contributions to overall fitness. By doing so, we may further identify traits that can be adaptively refined, and potentially even incorporated into novel phenotypes, following the expression of cryptic genetic variation.

## Author Contributions

**C. Hal Terry:** conceptualization, methodology, investigation, analysis, writing (original draft and revisions). **Dante J. Nesta:** conceptualization, methodology, investigation, writing (original draft and revisions). **Lucia Caluseriu:** investigation, writing (revision). **Meredith Pearson:** investigation, writing (revision). **Cristina C. Ledón‐Rettig:** conceptualization, methodology, investigation, analysis, writing (original draft and revisions).

## Conflicts of Interest

The authors declare no conflicts of interest.

## Supporting information


**Supplemental Figure 1:** Heritability estimates of snout vent length (top) and residual tail depth (bottom) using all individuals across microcosms. **Supplemental Table 1:** Point estimates and 95% confidence intervals of heritability. Descriptions of coefficient of genetic variation calculation and results **Supplemental Table 2:** Coefficients of genetic variation estimated from morphological measures bootstraps. **Supplemental Table 3:** Point estimates of 95% confidence intervals of coefficients of genetic variation.

## Data Availability

The data that support the findings of this study are available from the corresponding author upon reasonable request.
