# Supplementary material for: Competition's Role in Shaping Cryptic Genetic Variation
Source: Evol Dev. 2026 Mar 1;28(1):e70033. doi: 10.1111/ede.70033 (PMC13273344; doi:10.1111/ede.70033)
Supplement: Supplementary file 1 — Supplemental Figure 1: Heritability estimates of snout vent length (top) and residual tail depth (bottom) using all individuals across microcosms. Supplemental Table 1: Point estimates and 95% confidence intervals of heritability. Descriptions of coefficient of genetic variation calculation and results Supplemental Table 2: Coefficients of genetic variation estimated from morphological measures bootstraps. Supplemental Table 3: Point estimates of 95% confidence intervals of coefficients of genetic variation. [file EDE-28-e70033-s001.docx]

**Supplementary Material**

**Competition's Role in Shaping Cryptic Genetic Variation**

**This document includes the following:**

**Supplemental Table 1:** Point estimates and 95% confidence intervals of heritability.

Descriptions of coefficient of genetic variation calculation and results

**Supplemental Table 2**: Coefficients of genetic variation estimated from morphological measures bootstraps.

**Supplemental Table 3**: Point estimates of 95% confidence intervals of coefficients of genetic variation.

**Supplemental Figure 1:** Heritability estimates of snout vent length (top) and residual tail depth (bottom) using all individuals across microcosms.

|  | Low | | High | | |
| --- | --- | --- | --- | --- | --- |
| Trait | Detritus | Shrimp | | Detritus | Shrimp |
| **SVL** | **0.074** (0-0.43) | **0.032** (0-0.340) | | **0.101** (0-0.636) | **0.256** (0-0.7014) |
| **GL** | **0.458** (0.014-1.127) | **0.242** (0.002-0.677) | | **0.268** (0.0004-0.723) | **0.774** (0.04-1.26) |
| **JA** | **0.174** (0-1.23) | **0** (0-0.430) | | **0** (0-0.158) | **0** (0-0.698) |
| **TD** | **0.006** (0-0.431) | **0.031** (0-0.340) | | **0.027** (0-0.637) | **0.026** (0-0.701) |

Supplemental Table 1: Point estimates (**bold**) and 95% confidence intervals of heritability. Estimates of variation from the linear mixed model were used for the point estimates, while bootstrapped variances (10000 permutations) were used to calculate 95% confidence intervals.

**Coefficient of genetic variation analyses**

**Methods**: In addition to the comparison of heritability values, we also calculated the coefficient of genetic variation, another estimator of a trait’s potential response to selection (Houle, 1992). To calculate the coefficient of genetic variation, we used the following equation,

$$CVG=\frac{\sqrt{V_{AF}}}{x̄}*100$$

where $V_{AF}$ as among-family variance and x̄ representing the trait mean. It should be noted we used the non-standardized trait values; we used the following equation to account for the effect of centered size on the response trait.

$$Response trait=1+centered mean*diet*density\left( 0+diet:density | family \right)+\left( 1 | Microcosm \right)$$

**Results**: SVL and gut length followed similar patterns to that of our heritability estimates, as the shrimp diet decreased coefficients of genetic variation under low competition, but then increased under high competition (Table 2). The coefficient of genetic variation for jaw area decreased with the shrimp diet for both high and low competition, while the residual tail depth coefficient of genetic variation increased under both density treatments. The different patterns of trait values (heritability and the coefficient of genetic variation) across diet and density treatments are likely due to environmental variance not being used in the calculation of the latter trait value (Houle, 1992; Roff, 2012).

|  | Low | | | High | | |
| --- | --- | --- | --- | --- | --- | --- |
| Trait | Detritus | Shrimp |  | Detritus | Shrimp | 95% CI |
| SVL | 1.578% | 1.412% | <2.2e-16 | 1.537% | 2.701% | <2.2e-16 |
| GL | 9.080% | 4.704% | < 2.2e-16 | 6.690% | 11.121% | <2.2e-16 |
| JA | 12.193% | 3.480% | <2.2e-16 | 4.132% | 2.943% | <2.2e-16 |
| TD | 3.620% | 3.964% | <2.2e-16 | 4.661% | 4.932% | 4.863e-05 |

Supplemental Table 2: Different diet and density treatments influence the coefficient of genetic variation. It should be noted that these estimates were derived from the original morphological measures, not the size-corrected residuals. As with the heritability estimates, we used a Bonferroni correction to account for multiple tests (n=8), resulting in significance threshold of (p<0.00625).

| Trait | Detritus | Shrimp | Detritus | Shrimp |
| --- | --- | --- | --- | --- |
| SVL | 1.501 (0-3.847) | 1.018 (0-3.378) | 1.565 (0-3.314) | 3.098 (0-5.379) |
| GL | 9.354 (0-18.794) | 4.273 (0-10.344) | 7.649 (0-12.623) | 1.366 (0-21.471) |
| JA | 11.742 (0-23.220) | 2.142 (0-10.094) | 0 (0-12.861) | 3.049 (0-8.865) |
| TD | 0.657 (0-8.90) | 4.4189 (0-8.340) | 4.613 (0-10.277) | 1.120 (0-11.923) |

Supplemental Table 3: Point estimates (**bold**) and 95% confidence intervals of coefficients of genetic variation from the original morphological measures. As with the heritability calculation, estimates of variation from the linear mixed model were used for the point estimates, while variances from the bootstrapped model (10000 permutations) were used to calculate 95% confidence intervals.

Figure 1: Heritability estimates of snout vent length (top) and residual tail depth (bottom) using all individuals across microcosms. The brackets indicate significant differences of estimated heritability (p<2.2e-16). Patterns of heritability for SVL was consistent with prior assessments using only the largest individual per microcosm, while tail depth heritability decreased under both low and high competition with a novel shrimp diet. The reduced level of heritability is likely attributed to more variation across individuals from each microcosm.

**Works Cited**

Houle, D. 1992. Comparing evolvability and variability of quantitative traits. *Genetics* 130: 195–204.

Roff, D.A. 2012. Evolutionary quantitative genetics (Springer Science & Business Media).
